# Unwanted sexual attention at work and long-term sickness absence: a follow-up register-based study

**DOI:** 10.1186/s12889-016-3336-y

**Published:** 2016-07-30

**Authors:** Annie Hogh, Paul Maurice Conway, Thomas Clausen, Ida Elisabeth Huitfeldt Madsen, Hermann Burr

**Affiliations:** 1 Department of Psychology, University of Copenhagen, Øster Farimagsgade 2A, 1353 Copenhagen, Denmark; 2 National Research Centre for the Working Environment, Lersoe Parkalle 105, 2100 Copenhagen, Denmark; 3 Federal Institute of Occupational Safety and Health, Nöldnerstraße 40-42, 10317 Berlin, Germany

**Keywords:** Sexual harassment, Unwanted sexual attention, Long-term sickness absence, Gender differences, Bullying

## Abstract

**Background:**

The current understanding of the relationship between unwanted sexual attention at work and long-term sickness absence (LTSA) is limited for three reasons: 1) the under-researched role of unwanted sexual attention perpetrated by individuals outside the work organization; 2) a widespread use of self-reported measures of sickness absence, with an unclear identification of sickness absence episodes of long duration; 3) the cross-sectional design of most existing studies. The aim of this study was therefore to investigate the relationship between self-reported unwanted sexual attention at work and subsequent LTSA (≥3 weeks), stratifying by gender and source of exposure (i.e., colleagues, managers and/or subordinates vs. clients/customers/patients).

**Methods:**

This prospective study is based on a pooled sample of 14,605 employees from three Danish surveys conducted in 2000, 2004 and 2005, providing a total of 19,366 observations. A single questionnaire-based item was used to assess exposure to unwanted sexual attention. The pooled dataset was merged with Danish register data on LTSA. The risk of first-onset episode of LTSA (up to 18 months after baseline) in connection with unwanted sexual attention was examined using Cox proportional hazards models. We estimated Hazard ratios (HR) and 95 % confidence intervals (95 % CI) adjusted for age, influence at work, work pace, occupational group and mode of data collection. We also adjusted for repeated measures from individual respondents by stratifying the Cox models by wave of survey.

**Results:**

Unwanted sexual attention from colleagues, managers and/or subordinates predicted LTSA among men (HR 2.66; 95 % CI 1.42-5.00). Among women, an elevated but non-statistically significant risk of LTSA (HR 1.18; 95 % CI 0.65-2.14) was found. Unwanted sexual attention from clients/customers/patients did not predict LTSA, neither among men nor among women.

**Conclusions:**

The findings indicate a significantly elevated risk of LTSA, among men only, in relation to exposure to unwanted sexual attention from colleagues, managers and/or subordinates. This study therefore suggests both individual and organizational costs associated with unwanted sexual attention at work. Due to the low prevalence of unwanted sexual attention, larger studies with more statistical power are needed to confirm (or disconfirm) the present findings.

## Background

It is widely recognized that long-term sickness absence bears high costs to the employees, the work organizations and the society at large [1, 2]. Long-term sickness absence predicts severe outcomes such as work disability and labour market exclusion [3]. A priority for research is therefore to identify potentially preventable workplace risk factors of long-term sickness absence. The present study aims to contribute in this direction by investigating the under-researched role of being exposed to unwanted sexual attention at work, a common form of workplace sexual harassment [4], as a risk factor for long-term sickness absence (≥3 weeks). This relationship will be examined stratified by gender and for two different sources of unwanted sexual attention, i.e., colleagues, managers and/or subordinates or clients/customers/patients.

Workplace sexual harassment can be defined as unwelcome sex-related behaviours at work that are appraised by targets as offensive, exceeding their resources and threatening their well-being [5–7]. Such harassment may also reflect a specific form of workplace bullying in which gender or sexuality is utilized as a means of oppression [8]. According to the latest European Working Condition Survey (EWCS), workplace sexual harassment has an average prevalence of about 1 % among workers in EU27, and it does not exceed 2 % in any of the participating EU Member States, although these figures might be heavily underestimated because of underreporting bias. Exposure to sexual harassment is more prevalent among women and in sectors where employees are more in contact with clients or customers, such as healthcare, wholesale and retail [9, 10].

Specifically, unwanted sexual attention has been identified as the second most common form of workplace sexual harassment after gender harassment [6, 11, 12], which indicates sexist behaviour or acts aimed at belittling or offending the target on grounds of gender. The third form of sexual harassment is sexual coercion, which is more rare [11]. Unwanted sexual attention consists of sex-related verbal and/or non-verbal acts that are qualified as unwelcome, offensive and unreciprocated [11], such as sending intrusive letters, making phone calls, touching, grabbing, cornering, etc. [4]. In the available literature unwanted sexual attention has rarely been investigated on its own [13]. Thus, the following section refers to earlier literature on workplace sexual harassment in general, unless otherwise stated.

Previous cross-sectional evidence suggests that exposure to workplace sexual harassment is associated with poor health status and well-being [13, 14]. Health correlates of sexual harassment include negative mood and poor psychosomatic health [15, 16], symptoms of post-traumatic stress disorder [17], physiological stress [18], and depression [5]. Longitudinal studies on the association between sexual harassment on the one side and health and work-related consequences on the other side are less frequent but have linked sexual harassment with depressive symptoms [19], psychological distress [20], job dissatisfaction [21, 22], alcohol consumption [23], increased use of spiritual and legal services [24] and increased turnover [25].

Regarding sickness absence as a potential outcome, recent cross-sectional studies have found statistically significant associations between workplace sexual harassment and sickness absence of various length, although these associations were often weakened or became statically non-significant after adjustment for individual and work-related factors [26, 27]. Other older studies have also found statistically significant cross-sectional (e.g. [15, 28] and longitudinal (e.g. [21]) associations between workplace sexual harassment and work withdrawal, an unspecific outcome comprising a mixture of behaviours such as absenteeism, tardiness, and task avoidance. To our knowledge, the only available evidence regarding the specific prospective association between unwanted sexual attention and long-term sickness absence is provided by the studies conducted by Clausen et al. [29] on a sample of female Danish elder-care workers, and by Nabe-Nielsen et al. [30] on a workplace-based sample covering different occupational sectors . In the study by Clausen et al., the authors did not find a statistically significant effect of unwanted sexual attention on subsequent register-based long-term sickness absence of eight weeks or more [29]. On the contrary, a statistically significant association was found by Nabe-Nielsen et al. between unwanted sexual attention and later register-based long-term sickness absence of ≥30 days.

However, when examining the available literature at least three major limitations impact the current understanding of the relationship between unwanted sexual attention and long-term sickness absence. A first limitation lies in the way sickness absence has been assessed. Except for the studies by Clausen et al. [29] and Nabe-Nielsen et al. [30], the previously mentioned studies [26, 27] measured sickness absence using self-reports, while also failing to clearly identify sickness absence episodes of long duration. In these studies, sickness absence was included as outcome either in a continuous [26] or categorized form, by using yes / no absence [27] or >7 days of absence [31]. As a result, knowledge about the specific impact of unwanted sexual attention on long-term sickness absence is currently limited.

A second limitation is the cross-sectional design of most existing literature (see again Clausen et al. [29] and Nabe-Nielsen et al. [30] as rare exceptions). Because of this, the direction of the relationship between workplace sexual harassment and long-term sickness absence is unclear; indeed, a competing interpretation could be that employees with more sickness absence, because of their lower health status, generally have less resources and are thus more vulnerable to environmental demands, making them at a higher risk of being the target of sexual harassment and/or of perceiving certain acts as offensive and degrading.

The third limitation is that most existing research focuses on workplace sexual harassment perpetrated by co-workers [32, 33], although it may originate also from individuals not employed by the organization. In particular, there are no studies about the effect of such “third party” sexual harassment on long-term sickness absence. Such relationship could be expected given that employees in the service sector are often subjected to a “customer is always right” organizational culture [33] requiring them to maintain a sustained emotional regulation so as to conceal or limit the expression of their natural affective states even in front of harassing service users. Exposure to high demands for hiding emotions has previously been found to predict long-term sickness absence [34, 35]. No distinction between the two sources of unwanted sexual attention were made neither in the study by Clausen et al. [29] (although here unwanted sexual attention originated predominately from clients and patients [36] nor in the study by Nabe-Nielsen et al. [30]. This picture indicates that there is insufficient evidence about the potential differences in the association between unwanted sexual attention and long-term sickness absence related to the source of exposure (i.e., colleagues, managers and/or subordinates vs. clients/customers/patients).

A further open question in the current literature is whether the relationship between unwanted sexual attention and long-term sickness absence changes based on gender. Most existing research on workplace sexual harassment has been conducted on female employees, where the exposure is commonly more frequent [7]. Nevertheless, recent studies show that also men are subjected to this behaviour (i.e. [7, 12, 13, 15, 26, 27, 37]. However, the available studies provide mixed findings regarding the role of gender in the association between workplace sexual harassment and sickness absence [15, 26, 27, 37]. Hence, to date there is inconclusive evidence about whether the effect of unwanted sexual attention on long-term sickness absence differs between genders.

The present study aims to contribute addressing these limitations by investigating prospectively the relationship between exposure to unwanted sexual attention and the risk of long-term sickness absence (≥3 weeks) measured using registers. This association will be examined stratifying by gender and the source of unwanted sexual attention, i.e., from colleagues, managers and/or subordinates vs. clients/customers/patients.

## Methods

### Procedure and participants

This study is based on a pooled set of data from three existing Danish studies, i.e., the Danish Work Environment Cohort Study (DWECS-2000 (*N* = 5766) [38], the DWECS-2005 (*N* = 8901) [39]), and the Copenhagen Psychosocial Questionnaire Study (COPSOQ-II (*N* = 4669) [40]. These studies were linked to the Danish DREAM-register which contains information on all social transfer payments in Denmark, including sickness absence benefits since 1996 [41]. The COPSOQ-II and the two DWECS studies include samples that were drawn randomly from the Danish population of working age. Response rates to these surveys varied between 75 % (DWECS 2000), 60 % (COPSOQ-II 2004), and 63 % (DWECS 2005). The pooled sample used in the present study consisted of 19,366 observations deriving from 14,605 independent participants across the three surveys. This means that some employees participating in both DWECS surveys contributed twice to the data.

### Outcome: Long-term sickness absence

Long-term sickness absence (LTSA) was measured in the Danish DREAM-register on social transfer payments. In DREAM, sickness absence is recorded at a weekly basis when the employer is entitled to reimbursement of the sickness pay during our 18-month follow-up period. During the years in which this study took place (2000 to 2007), Danish employers were entitled to reimbursement after 14 consecutive days of sickness absence [42]. Therefore, we defined long-term sickness absence as absence exceeding 14 calendar days, corresponding to at least three consecutive weeks in DREAM (register codes 890–899). No diagnostic information on the causes of sickness absence is included in the DREAM register.

### Independent variable: Unwanted sexual attention

Unwanted sexual attention was operationalized with the following item: ‘Have you been exposed to unwanted sexual attention within the past 12 months?’, without a definition of the phenomenon provided ahead of the question. In DWECS-2000 and DWECS-2005, the response options were: ‘No’, ‘Yes, from colleagues’, ‘Yes, from a manager’, ‘Yes, from a subordinate’, and ‘Yes, from clients/customers/patients’.

In COPSOQ-II, the response options were: ‘Yes, daily’, ‘Yes, weekly’, ‘Yes, monthly’, ‘Yes, now and then’, and ‘No’. Respondents reporting exposure to unwanted sexual attention were subsequently asked who the perpetrator was. Response options were: ‘Colleagues’, ‘A manager’, ‘Subordinates’, and ‘Clients/customers/patients’ [40].

To harmonize measures across the three surveys, we recoded the item concerning unwanted sexual attention into two separate variables as follows:

- Self-reported exposure to unwanted sexual attention from colleagues, managers and/or subordinates (yes/no).
- Self-reported exposure to unwanted sexual attention from clients/customers/patients (yes/no).

### Covariates

Elements of the psychosocial work environment such as low influence at work and high work pace, have been shown to predict long term sickness absence [34, 43–46]. Poor psychosocial environment is associated with negative acts in general [47]. Thus, such work environment factors could potentially confound the association between unwanted sexual attention and long term sickness absence. *Influence at work* was measured using a four-item scale from COPSOQ-II [40]. Sample item: “Do you have a large degree of influence concerning your work?”. Cronbach’s alpha: 0.78. *Work pace* was measured using a single item from COPSOQ-II: “Is it necessary for you to work very fast?”. Responses were given on five point Likert-scales. The above-mentioned scale and single-item question on psychosocial work conditions were scored from 0–100, with 100 representing the highest degree of the measured dimension. Age and gender of respondents were derived from registers. Respondents were subdivided into five mutually exclusive occupational groups (working with customers, working with clients, office workers, manual workers and primary sector work [agriculture, forestry, fishing]) based on information on job titles from the original datasets. Finally, we also included as covariate a categorical variable identifying the different modes of data collection used in the three surveys, i.e., face-to-face interview, telephone interview, postal and on-line questionnaire, as this factor has been shown to affect the level of reporting [48].

### Statistical analysis

To investigate the effect of unwanted sexual attention on the risk of LTSA during the 18-month follow-up, data were analysed using Cox proportional hazards model. Hazard ratios (HR) and 95 % confidence intervals (95 % CI) were estimated. The risk time was calculated as time from answering the questionnaire until the first onset of sickness absence episode or the end of the 18 month follow-up period. Respondents who were cases of LTSA at baseline and those who emigrated, retired or died during the follow-up period, were censored from the study. The analyses were conducted separately for men and women distinguishing between the source of sexual harassment. Note that we did not exclude office workers, manual workers and primary sector workers from the analyses on unwanted sexual attention from clients/customers/patients and LTSA. We did so because also in these groups contacts with client and customers may occur. This is confirmed in our study, where unwanted sexual attention from clients/customers/patients was also reported by these workers. All analyses were cumulatively adjusted in three steps. In the first step, we adjusted for age and mode of data collection. In the second step, we additionally adjusted for occupational group. In the third step, we additionally adjusted for influence at work and work pace. We also stratified the Cox models by the original study, to account for data clustering within each study. This approach is similar to a fixed effect meta-analysis of the study-specific hazard ratios [49]. We included two waves of the DWECS study in the analyses and adjusted for repeated measures from individual respondents by stratifying the Cox models by wave of survey (i.e. DWECS2000 and DWECS2005) [50]. Finally, in a sensitivity analysis we excluded respondents who had been exposed to unwanted sexual attention from clients/customers/patients from the analysis of the association between unwanted sexual attention from colleagues, managers and/or subordinates and the risk of LTSA, and vice versa. Data were analysed using SAS 9.3 (SAS Institute Inc., Cary, NC, USA).

## Results

Table 1 shows the descriptive statistics for the main study variables. In all, unwanted sexual attention was reported by 2.2 % of the sample (2.9 % among women and 1.6 % among men). In particular, 1 % of the respondents were exposed to unwanted sexual attention from colleagues, managers and/or subordinates (1.3 % among women and 0.7 % among men); 1.3 % reported exposure from clients/customers/patients (1.7 % among women and 1.0 % among men). We note that unwanted sexual attention from clients/customers/patients was also reported by office workers (0.4 %), manual workers (0.4 %) and primary sector workers (0.6 %), although to a lower extent than those participants working with customers (2.1 %) and clients (2.7 %).

**Table 1:** Sample characteristics (overall and stratified by gender)

	All respondents	Female respondents	Male respondents
Observations (N /Per cent)	19,366 / 100.0	9599 / 49.6	9767 / 50.4
Long-term sickness absence (Events /Per cent)	1769 / 9.1	947 / 9.9	822 / 8.4
Exposure to unwanted sexual attention: Overall prevalence (N /Per cent)	415 / 2.2	265 / 2.9	150 / 1.6
Exposure to unwanted sexual attention from colleagues/managers/subordinates (N /Per cent)	181 / 1.0	118 / 1.3	63 / 0.7
Exposure to unwanted sexual attention from clients/customers/patients (N /Per cent)	245 / 1.3	156 / 1.7	89 / 1.0
Influence at work (Mean (SD))	52.8 (26.1)	50.4 (25.2)	55.1 (26.8)
Work pace (Mean (SD))	57.8 (24.6)	58.4 (24.7)	57.2 (24.5)
Age (Mean (SD))	41.4 (11.1)	41.4 (11.0)	41.5 (11.1)
Working with customers	2510 / 13.0	1378 / 14.4	1132 / 11.6
Working with clients	5825 / 30.1	3619 / 37.7	2206 / 22.6
Office workers	4831 / 25.0	2556 / 26.6	2275 / 23.3
Manual workers	5617 / 29.0	1843 / 19.2	3774 / 38.6
Primary sector work (agriculture, forestry, fishing)	583 / 3.0	203 / 2.1	380 / 3.9
Original survey (N /Per cent)			

In Table 2, gender-stratified Hazard Ratios (HR) indicated that exposure to unwanted sexual attention from colleagues, managers and/or subordinates was significantly associated with subsequent long-term sickness absence among men but not among women after adjusting for age and occupational group (HR = 2.15; 95 % CI = 1.18 - 3.91). Including influence at work and work pace in the third model did not substantially change the associations (HR = 2.47; 95 % CI 1.32 – 4.65). In the sensitivity analysis, when excluding respondents who had been exposed to unwanted sexual attention from clients/customers/patients, the HR remained significant (=2.66 95 % CI 1.42 – 2.66) (not shown in the table) among men. With regard to women, we found a slightly elevated but statistically non-significant HR for the association between unwanted sexual attention from colleagues, managers and/or subordinates and long-term sickness absence in the final fully adjusted model (HR = 1.10; 95 % CI = 0.60 - 2.00), which was also confirmed in the sensitivity analysis where participants also reporting unwanted sexual attention from clients, customers and/or patients were removed from the analysis (HR = 1.18; 95 % CI = 0.65 - 2.14) (not shown in the table).

**Table 2:** Risk of long-term sickness absence in relation to unwanted sexual attention from colleagues/managers/subordinates^a^

				Risk of LTSA for more than three consecutive weeks					
				Model 1^b^		Model 2^c^		Model 3^d^	
Gender	Exposed	Cases	Events n/%	HR	95 % CI	HR	95 % CI	HR	95 % CI
Female	Yes	118	12 / 10.2	1.17	0.66-2.08	1.21	0.68-2.15	1.10	0.60-2.00
	No	8978	890 / 9.9	1	Reference	1	Reference	1	Reference
Male	Yes	63	11 / 17.5	2.11	1.16-3.84	2.15	1.18-3.91	2.47	1.32-4.65
	No	9343	782 / 8.4	1	Reference	1	Reference	1	Reference

^a^ All estimates are adjusted for random effects from the original surveys that the pooled data are based upon and repeated measures from individual respondents

^b^ Hazard ratios are adjusted for age and mode of interviewing

^c^ Hazard ratios are additionally adjusted for occupational group

^d^ Hazard ratios are additionally adjusted for psychosocial work conditions (influence at work and work pace)

Table 3 shows an elevated but non-significant risk (HR = 1.31, 95 % CI = 0.67 – 2.54) for the association between unwanted sexual attention from clients/customers/patients and long-term sickness absence in men, while no such indication of an elevated risk was observed among women. Similar results were found in the sensitivity analysis excluding those participants also reporting unwanted sexual attention from colleagues, managers and/or subordinates.

**Table 3:** Risk of long-term sickness absence in relation to unwanted sexual attention from clients/customers/patients^a^

				Risk of LTSA for more than three consecutive weeks					
				Model 1^b^		Model 2^c^		Model 3^d^	
Gender	Exposed	Cases	Events n/%	HR	95 % CI	HR	95 % CI	HR	95 % CI
Female	Yes	156	14 / 9.0	0.96	0.56-1.63	0.89	0.52-1.51	0.89	0.52-1.51
	No	8940	888 / 9.9	1	Reference	1	Reference	1	Reference
Male	Yes	89	11 / 12.4	1.54	0.85-2.80	1.51	0.83-2.75	1.31	0.67-2.54
	No	9317	782 / 8.4	1	Reference	1	Reference	1	Reference

^a^ All estimates are adjusted for random effects from the original surveys that the pooled data are based upon and repeated measures from individual respondents

^b^ Hazard ratios are adjusted for age and mode of interviewing

^c^ Hazard ratios are additionally adjusted for occupational group

^d^ Hazard ratios are additionally adjusted for psychosocial work conditions (influence at work and work pace)

## Discussion

We found that self-reported exposure to unwanted sexual attention from colleagues, managers and / or subordinates was related to a higher risk of subsequent long-term sickness absence than exposure to unwanted sexual attention from clients/customers/patients. However, men were found to be more at risk of long-term sickness absence than women in connection with the exposure to unwanted sexual attention from colleagues, managers and / or subordinates. No statistically significant associations were observed among female employees and in relation to unwanted sexual attention from clients/customers/patients. These results were confirmed in sensitivity analyses excluding employees reporting simultaneous exposure to the two sources of unwanted sexual attention.

We must, however, note that, despite the large sample size, the results of the present study might have been affected by limited statistical power. A confidence interval-based power calculation using the estimates in Tables 2 and 3 (for this method of power calculation see Pejtersen et al. [51]), shows that the present study has an 80 % chance of detecting, as statistically significant, a HR of 2.5 or more. This means that, although we were able to find statistically significant HRs lower than 2.5, a replication of the study would have less than 80 % chance of detecting as statistically significant a true HR lower than 2.5, given similar prevalence estimates and distributions of exposure and outcome. The low power is mainly due to the low prevalence of the exposure, which was 2.2 % overall and 1 % and 1.3 % for unwanted sexual attention from colleagues, managers and/or subordinates and from clients/customers/patients, respectively. Although low, these prevalence estimates are however in line with those observed in the European Working Condition Survey [9]. Therefore, the interpretations we provide below of the study findings need to be confirmed in future studies based on larger samples.

With the present study, we sought to address some of the pitfalls in previous research limiting the knowledge of the potential link between unwanted sexual attention and long-term sickness absence. Most available studies adopted self-reported measures of sickness absence while failing to clearly identify employees with sickness absence of long duration, used cross-sectional designs and made no distinctions between different sources of workplace sexual harassment. We are aware of only two prospective studies that examined the relationship between unwanted sexual attention and long-term sickness absence [29, 30]. Clausen et al. [29] found a statistically non-significant association between unwanted sexual attention and subsequent LTSA of eight weeks or more. However, Clausen et al.’s study was conducted on a single occupation (the elder-care sector) and among female workers only, and did not specify the source of unwanted sexual attention, despite in their sample most of the negative acts were perpetrated by clients/patients. Nabe-Nielsen et al. [30] found a significant association between unwanted sexual attention and LTSA in a sample composed by various occupational groups but without stratifying the analyses by gender and source of exposure. By contrast, in our study based on a representative sample of the Danish working population, we focused on potential differences related to gender and source (i.e., by colleagues, managers and/or subordinates or clients/customers/patients) while examining the association between unwanted sexual attention and long-term sickness absence.

The statistically significant effect of unwanted sexual attention by colleagues, managers and/or subordinates on long-term sickness absence may be explained in the light of the “Stress-as-Offence-to-Self” theory [28]. This theory posits that social stressors (also referred to as “illegitimate behaviours”), which include negative acts such as workplace bullying and sexual harassment, are likely to constitute a threat to one’s self-esteem because they signal lack of appreciation and respect and frustrate one’ sense of belonging to a significant group. Low self-esteem represents one of the most important pathways of the association between adverse psychosocial working conditions and mental health problems [52], the latter being a predictor of long-term sickness absence [53].

In light of the theoretical framework of personal control proposed by Thacker [54], targets may be expected to use short spells of sickness absence as a way to recover and be ready to meet future possibly unpleasant situations, in this way regaining personal control of an adverse situation like being subjected to unwanted sexual attention. However, Knorz and Zapf (in Zapf and Gross [52]) found in a qualitative study that what they called successful copers, among other things, took longer times out (long-term sick leave) to get some personal stabilization compared to the unsuccessful copers who took frequent short-term leaves.

### Gender differences

The results of previous cross-sectional studies are mixed regarding gender differences in the association between unwanted sexual attention and sickness absence (26;27;31). In one study, the association between workplace sexual harassment and long-term sickness absence was stronger among women than men [31], although the link became non-significant for both genders when adjusting for potential confounders. However, two other studies found that this association was similar across genders [26] or even stronger among men [27]. Studies focusing on health effects of workplace sexual harassment that might operate as potential risk factors for long-term sickness absence found the same mixed evidence (e.g., [20, 23, 24, 55]). Therefore, as yet, research has failed to establish any clear gender difference in the association between workplace sexual harassment and sickness absence.

In our study we found a statistically significant association between unwanted sexual attention from co-workers, managers and/or subordinates and long-term sickness absence among men only. This finding may be interpreted through the lens of the previously mentioned “Stress-as-Offence-to-Self” theory [28]. Men may differ from women in the way they perceive and cope with unwanted sexual attention, which might affect the potential (health) consequences associated with this exposure. In particular, male employees might perceive being a target of sexual harassment from co-workers as an even bigger threat to their self-esteem than it is for their female counterparts [7]. This may occur because in our society traumas of sexual nature are generally viewed as less accepted among men, with consequences on the perception of one’s gender role identity being potentially more severe for men than for women [55]. The shame that results from being exposed to unwanted sexual attention may lead men to avoid talking about the problem and taking actions to fix the situation, thus reducing the effectiveness of their problem-solving and/or emotional coping behaviours [56]. This may also interact with a generally lower tendency among men to seek support in the face of difficult events as compared to women [57]. For instance, men may be less inclined than women to make use of health services [54], reducing the possibility for them to prevent the onset or worsening of mental and/or physical health problems when exposed to adverse circumstances.

Another potential explanation for the observed gender differences might be that women have a lower threshold for labelling a behaviour as sexual harassment [58]; therefore, it might be that the experiences described as unwanted sexual attention are perceived as more severe among men than women, thus leading to worse effects on health among the former.

### Differences related to the source of unwanted sexual attention

Contrary to most previous research, in our study we considered the effect on long-term sickness absence of two sources of unwanted sexual attention separately. In addition to the inclusion of both men and women, this distinction could explain the different results between ours and Clausen et al.’s [29] study, where the authors did not distinguish between source of exposure and the major part of negative behaviours were perpetrated by clients/customers/patients [36]. Although also sexual harassment from external clients is linked with reduced health and well-being [32, 33], one might expect, again in line with the “Stress-as-Offence-to-Self” framework, that being sexually harassed by co-workers is considered by employees – even more so by men, as discussed previously - as more illegitimate and threatening to their self-esteem than when the perpetrators are clients/customers/patients. To a certain degree, employees may view the risk of being confronted with deviant behaviours by external users, including sexual attentions, to be intrinsically connected to their role. In addition, facing sexually harassing customers may constitute an experience which is shared by employees holding similar jobs in organizations [33]. Consistent with the social identity theory [59], this may reinforce the perceived distance between the internal and the external group (i.e., the service users) and strengthen an employee’s sense of belonging to his/her group at work. Social identification may thus promote collective ways of coping and therefore act as a protective factor against the negative consequences of being exposed to unwanted sexual attention by customers, clients and/or patients.

### Strengths and limitations

The present study has three major strengths. First, it presents good external validity since the population examined is based on large, representative and randomly selected samples of the adult Danish working population. In addition, the response rates obtained across the surveys were fairly high for this type of study (>60 %). Second, internal validity is supported by the use of a prospective design and two different sources of measurement for the assessment of the exposure (i.e., self-reports) and the outcome (i.e., register-based long-term sickness absence). Register data are more reliable than self-reports when measuring sickness absence. They reduce the impact of common method variance and allow almost all participants to be followed-up. A third strength is that we distinguished between two separate sources of unwanted sexual attention, which is unprecedented in studies on the association between workplace sexual harassment and long-term sickness absence.

However, our study also presents limitations. The major one relates to the above-mentioned limited statistical power. Low power additionally prevented us from formally testing the interaction between gender and exposure on the outcome. Therefore, even though the HRs for the association between unwanted sexual attention and long-term sickness absence differed among men and women in both the main and the sensitivity analyses, we cannot draw definite conclusions about whether the examined relationship is actually modified by gender. Studies with even larger sample sizes are therefore needed to ascertain the existence of such interactions.

We used self-reports to measure unwanted sexual attention. The self-reporting of this exposure could be influenced by factors such as personal traits and/or the health status of the respondent. For instance, employees with a poorer mental health might over-report exposure to unwanted sexual attention, leading to a possible overestimation of the association between the latter and long-term sickness absence.

Another limitation was that the data we examined in this study was collected between 2000 and 2005 and might thus be considered not applicable to today’s situation. However, this may hold true particularly when focusing on the prevalence of a given phenomenon, which was not the case in our study. By contrast, examining older data might be seen as less of a problem when it comes to the investigation of relationships between phenomena which are not linked to certain time periods, as supported by the prevalence estimates from our study being similar to those observed in the recent EWCS survey [9].

## Conclusions

This study showed that being exposed to unwanted sexual attention at work has long-term consequences for the target in the form of a higher risk of long-term sickness absence among men exposed to unwanted sexual attention from co-workers, managers and/or subordinates. Apart from the negative implications this has for the employee, these findings may also indicate negative implications for organizations that tolerate unwanted sexual attention. In addition to creating a negative psychosocial climate, sickness absence may reduce organizational effectiveness and efficiency by increasing labour costs. Often substitutes are needed to cover for the absent employee, who in many occasions still has to get paid, resulting in increased costs for the organization [60]. Thus, employers in collaboration with employees should consider implementing strategies to prevent unwanted sexual attention at work at three levels: 1) organizational: i.e. developing a policy including focus on how to deal with sexually harassing behavior, management training; 2) job / task level: i.e. psychosocial work environment redesign, risk analysis, awareness training; 3) at the individual level: i.e. assertiveness training, social support [61].

There is, however, a need for more prospective studies measuring consequences of the three elements of sexual harassment: gender harassment, unwanted sexual attention and sexual coercion for both men and women using a specific definition of the exposure and criteria for collecting episodes at time-points that are able to capture both short- and long-term consequences. Given the typical low prevalence of unwanted sexual attention, future studies based on larger samples are needed to confirm (or disconfirm) the results of the present study. These studies may also distinguish between unwanted sexual attention perpetrated by colleagues/subordinates vs. managers. Finally, we suggest that future studies test the mediating role of low self-esteem in the relationship between sexual harassment / unwanted sexual attention, mental health problems and long-term sickness absence.
